# The Association Between Alcohol Use and Chronic Diseases’ Treatment Outcomes Among Adults Aged 40 Years and Above in Rural South Africa

**DOI:** 10.21203/rs.3.rs-3385716/v1

**Published:** 2024-02-23

**Authors:** Rumbidzai Mupfuti, Chodziwadziwa Kabudula, Joel Francis

**Affiliations:** University of the Witwatersrand; University of the Witwatersrand; University of the Witwatersrand

**Keywords:** Alcohol use, HIV, Hypertension, Diabetes Mellitus, Multimorbidity, Treatment Outcomes, Older Adults, South Africa

## Abstract

Chronic diseases are significant problems in South Africa. Chronic diseases’ treatment outcomes are critical to the reduction of morbidity and mortality. There is limited data in South Africa on alcohol use and treatment outcomes of chronic diseases in older people. We analysed data from wave 1 of the Health and Ageing in Africa-a longitudinal Study in an INDEPTH community (HAALSI) study. We performed descriptive analysis to determine the prevalence of optimal chronic diseases’ treatment outcomes (suppressed HIV viral load, normal blood pressure and normal blood sugar) and applied multivariate modified Poisson regression to determine the association between alcohol use and chronic diseases’ treatment outcomes. The prevalence of optimal treatment outcomes were 87.4% for HIV, 42.7% for hypertension, 53.6% for diabetes mellitus and 52.4% for multimorbidity. Alcohol use did not negatively impact the treatment outcomes for HIV (aRR=1.00, 95%CI:0.93–1.09), hypertension (aRR=0.88, 95%CI:0.68–1.14), diabetes mellitus (aRR=0.73, 95%CI:0.44–1.22), and multimorbidity (aRR=1.00, 95%CI:0.93–1.09). Alcohol use was not significantly associated with treatment outcomes possibly due to underreporting of alcohol use. There is need to incorporate objective alcohol measurements in chronic diseases care settings. Furthermore, there is urgent need to strengthen the management of hypertension and diabetes, by adopting the strategies deployed for HIV management.

## INTRODUCTION

South Africa faces a quadrupled burden of disease resulting from communicable, non-communicable diseases, maternal-child mortality, and injuries (1). This is due to the rapid epidemiological and nutritional transitions occurring because of improved healthcare and disease prevention strategies (2). HIV, hypertension, and diabetes mellitus are among the top prevalent chronic conditions causing mortality in South Africa, with the elderly population succumbing to multiple conditions resulting in multimorbidity. Multimorbidity which is the existence of two or more chronic conditions in the same individual (3), leads to clinical complexity.

Older adults tend to experience a disproportionate share of NCDs (non-communicable diseases) burden and related complications such as CVDs (cardiovascular diseases) (4). A demographic shift in PLHIV (people living with HIV) to older age has led to an increased risk of acquiring NCDs (5). Uncontrolled hypertension and diabetes mellitus are among the leading risk factors for CVDs (4). In addition, HIV-infected individuals are also at an increased risk for CVDs. There is a great need to prioritize treatment control strategies for chronic diseases as part of an initiative to prevent the growing incidence of CVD and premature mortality.

The rates of control of chronic diseases (HIV, hypertension, and diabetes mellitus) are still low in Africa, despite advances in treatment. According to the WHO SAGE wave 2, only 18% of people on antihypertensives were controlled (6). An estimated 19% of people on treatment for diabetes mellitus achieved glycemic control according to the SADHS 2016 (7). Conversely, according to the 2017 National Population-Based Survey about 87% of those on HIV antiretroviral treatment achieved viral suppression (8). Achieving treatment control is multifaceted, requiring the synergy of different prevention interventions.

Alcohol consumption has been recognized as a unique modifiable risk factor for population health, affecting both infectious and non-communicable diseases (9). Alcohol is a toxic and addictive drug that has been shown to play a role in some mechanisms suggesting a multiplicative effect in acquiring the chronic condition (10). The aging population is more susceptible to the toxic effects of alcohol, with the already increased chronic disease burden amongst this group. As of 2016, the percentage of alcohol-attributable deaths was 12.9% for infectious diseases and 19% for cardiovascular disease and diabetes worldwide with the highest age-standardized alcohol-attributable burden of disease and injury of 70.6 deaths per 100 000 people occurring within the African region (9). The 2016 SADHS reported problem drinking in 15% of men and 2.5% of women aged between 45 and 64 years old (11). Harmful alcohol use is an important public health concern that needs to be addressed among the older population to control chronic diseases.

A few studies have reported on the alcohol use and treatment outcomes for chronic diseases in an aging population in sub-Sahara Africa. The studies done have reported inconsistent findings. Some researchers reported an association between alcohol use and treatment outcomes for HIV, hypertension, and diabetes mellitus (12–14) while other researchers reported no association (15–17). Findings from this study will help to inform interventions targeted at improving treatment outcomes of chronic diseases (HIV, hypertension, and diabetes mellitus. The main aim of this study was to determine the association between alcohol use and treatment outcomes of HIV, hypertension, and diabetes mellitus among adults aged 40 years and above in rural South Africa.

## METHODS

### Study setting

The primary study was conducted in South Africa, Mpumalanga Province in a rural sub-district where the Agincourt Health and Demographic Surveillance Site (HDSS) has been in operation since 1992 (18). The Agincourt HDSS study area of approximately 450km^2^ is spread across thirty-one villages and covers a population of 116000 individuals (18).

We used data from wave 1 of the “Health and Aging in Africa: A Longitudinal Study of an INDEPTH Community in South Africa (HAALSI)” study. The baseline survey comprised men and women aged 40 years or older sampled from the ongoing Agincourt HDSS (18). The study was conducted between November 2014 and November 2015. Information on cognitive and physical functioning, social networks, economic well-being, cardiometabolic disease and HIV risk factors was collected through interviewer-administered questionnaires. Also included in the survey were anthropometric measurements and point-of-care blood tests for haemoglobin, glucose, and lipids. dried blood spots were collected and later tested for HIV, HIV viral load, and C-Reactive Protein (18).

### Study Design and Population

This is a cross-sectional study addressing the research question through secondary data analysis of the HAALSI dataset. There were 12875 men and women from the Agincourt HDSS who met the eligibility criteria of being aged 40 years and above and permanently residing in the Agincourt HDSS study site for 12 months preceding the 2013 census (18). From the sampling frame, 6281 were randomly chosen to participate with gender-specific sampling fractions being developed. However, 5059 people (2345 men and 2714 women) completed their interviews and were enrolled in the study (18). The eligibility criteria for our study is all participants aged 40 years and above in the 2014 HAALSI study who self-reported being on treatment for the following chronic diseases: HIV, hypertension, and diabetes mellitus.

### Outcomes

The main outcomes are optimal treatment outcomes for HIV, hypertension, diabetes mellitus and multimorbidity. Ascertainment of being on treatment was self-reported for hypertension, diabetes mellitus and HIV based on the questions: “*ever received treatment for high blood pressure?”, “ever received treatment for diabetes?” and “ever received ART?”* respectively. The optimal treatment outcomes is based on local South African guidelines. The optimal HIV treatment outcome (controlled) is defined as a viral load of less than 1000 copies/ml (19). The optimal treatment outcome (controlled) for hypertension is defined as systolic blood pressure < 140mmHg and diastolic blood pressure < 90mmHg (20). The optimal treatment outcome (controlled) for diabetes mellitus is defined as a random blood glucose of less than 11.1mmol/l or fasting blood glucose of less than 7mmol/l (21). Multimorbidity is defined as the existence of 2 or more chronic conditions from the following diseases (HIV, hypertension, and diabetes mellitus). The optimal treatment outcome for multimorbidity is defined as having optimal treatment outcomes for all 3 chronic diseases. Outcome variables were categorized as 0 = controlled and 1 = uncontrolled.

### Primary exposure

The main exposure is alcohol use which is defined according to the frequency of drinking alcohol in the past 30 days by the following question: *“How often do you have at least 1 alcoholic drink?”* The following responses are given: daily drinking, 5–6 days per week, 1–4 days per week, 1–3 days per month, less than once per month and does not currently drink. alcohol use was recategorized into 3 groups for HIV, hypertension, and multimorbidity treatment outcomes: at least once a week (daily drinking, 5–6 drinks per week and 1–4 days a week), at least once a month (1–3 drinks per month and less than 1 per month) and none (does not currently drink). For diabetes mellitus treatment outcome, alcohol use was categorized into 2 groups, 0 = none and 1 = Yes.

### Covariates

The covariates include age group, sex, education category, nationality, BMI, employment status, wealth asset index, marital status, size of the household and tobacco use which were self-reported. Age group was recategorized into 2 groups: “40–60 years” and “60 years and older” (22). Wealth index was created from household characteristics and asset ownership using the Principal Component Analysis.

### Data management and analysis

STATA version 17 has been used for data management and analysis (23). Descriptive statistics of the total study population have been computed for alcohol use and covariates. This is reported as frequencies and proportions. Pearson’s Chi-square test is used to determine the prevalence of optimal treatment outcomes for each chronic condition (HIV, hypertension, diabetes mellitus and multimorbidity) across alcohol use and other covariates. Each selected exposure variable is cross tabulated with the optimal treatment outcome of each chronic disease (HIV, hypertension, diabetes mellitus and multimorbidity). The output of this cross-tabulation is reported as proportions (n, %). (*Supplementary Tables* 1–5). Univariate modified Poisson regression is used to assess the relationship between the optimal treatment outcome of each chronic disease (HIV – viral load suppression, hypertension – controlled blood pressure, diabetes mellitus – controlled glycemia, and multimorbidity – optimal for all the three conditions) with alcohol use and the covariates. Then, all exposures with p-value (< 0.20) are added in the multivariate modified Poisson regression(24) to determine the association of alcohol use and each chronic disease treatment outcome adjusting for other factors. The final model also includes exposure variables considered as apriori confounders including sex, age, tobacco use and wealth index (12–14). Interaction terms among alcohol use and exposure variables (age and sex) are checked for all the four models and all are not statistically significant. The final model fit for the association between alcohol use and the chronic diseases’ treatment outcomes (HIV, hypertension, diabetes mellitus and multimorbidity) is assessed using a post-estimation goodness-of-fit test. Finally, adjusted relative risk (aRR) and their corresponding 95% confidence intervals (95% CI) and p values from the final models are reported. A p value of < 0.05 was considered statistically significant.

### Ethical considerations

The HAALSI study received ethics approval from the following institutions: The University of the Witwatersrand ref *M141159* (Appendix 4), the Harvard School of Public Health *ref C13–1608-02* and the Mpumalanga Provincial Office. The participants’ data were coded and de-identified. For this study, ethics approval was obtained from the University of the Witwatersrand Human Research Ethics Committee *ref M230446* (Appendix 3).

## RESULTS

### Population Characteristics

Out of the 5055 participants, almost three quarters (76.9%) reported no alcohol use with only 12.2% reporting alcohol use at least once a week and 10.9% reporting alcohol use at least once a month. More than half of the participants (53.7%) were female and 51% of participants were aged 60 years and above. 1526 (30%) of the participants within the study population were migrants. 2306 participants (45.7%) received no formal education and a third of the participants (34%) received some primary level of education. Just over half of the participants (50.9%) were married and participants were approximately equally distributed with an average of 20% of participants per wealth index quintile. Most of the participants (73.7%) were not working and 48.2% (n = 2438) of participants lived in a 3 to 6 people household. Lastly, the majority of the participants (n = 4608) reported no tobacco use and only 36.7% of participants had a normal BMI. (Table 1) Prevalence of HIV viral load suppression controlled (glycemia and blood pressure) and optimal outcomes for all conditions among those with multimorbidity.

**Figure 1:**
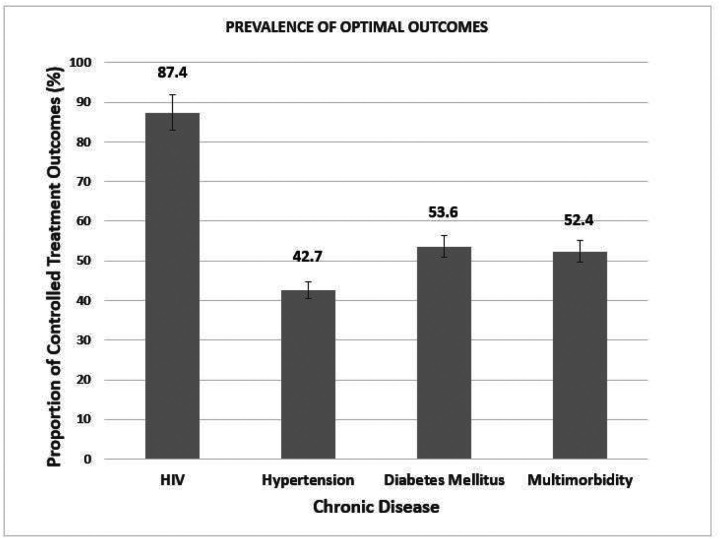
Prevalence of HIV viral load suppression, controlled (glycemia and blood pressure) and optimal outcomes for those with multimorbidity.

Figure 1 shows the prevalence of optimal chronic diseases’ treatment outcomes for HIV, hypertension, diabetes mellitus and multimorbidity. The prevalence of optimal treatment outcomes was 87.4% (450/515) HIV viral load suppression, 42.7% (713/1668) for controlled hypertension, 53.6% (140/261) for diabetes mellitus and 52.4% (475/907) for multimorbidity.

### Alcohol use and HIV viral load suppression

There was no statistical difference on HIV viral load suppression between participants reporting alcohol use at least once a week (aRR = 1.00, 95% C.I: 0.93–1.09) and those reporting alcohol use at least once a month (aRR = 0.92, 95% C.I: 0.8–1.05) compared to those that reported no alcohol use (Table 2).

### Other factors associated with HIV viral load suppression:

Females were 13% more likely to have HIV viral load suppression (aRR = 1.13, 95% C.I: 1.04–1.24) compared to males. Similarly, participants who reported to have attained secondary education or more were 14% more likely to have HIV viral load suppression (aRR = 1.14, 95% C.I: 1.01–1.21) compared to participants that reported no formal education. Overall, there was no association found between HIV treatment outcomes and the following covariates: age, wealth index, BMI, tobacco use and marital status. (Table 2)

### Alcohol use and controlled hypertension

Participants reporting alcohol use at least once a week and at least once a month were 12% less likely to have controlled hypertension (aRR = 0.88, 95% C.I: 0.68–1.14) compared to those that reported no alcohol use. However, the associations were not statistically significant. (Table 3)

#### Other factors associated with controlled hypertension:

Female participants were 22% more likely to have controlled hypertension (aRR = 1.22, 95% C.I: 1.07–1.39) compared to male participants. Participants aged above 60 years were 18% more likely to have controlled hypertension (aRR = 1.18, 95% C.I: 1.05–1.34) compared to participants aged between 40 and 60 years old. Conversely, obese (aRR = 0.75, 95% C.I: 0.64–0.84) and overweight (aRR = 0.84, 95% C.I: 0.73–0.97) participants were 25% and 16% less likely to have controlled hypertension respectively compared to participants with a normal BMI. There was no association between hypertension treatment outcomes and the following covariates: country of origin, wealth index and tobacco use. (Table 3)

### Alcohol use and controlled glycemia

Among those with diabetes, participants reporting alcohol use were 27% less likely to have controlled glycemia (aRR = 0.73, 95% C.I: 0.44–1.22) compared to those that reported no alcohol use. However, this association was not statistically significant. (Table 4)

#### Other factors associated with controlled glycemia:

Female participants were 25% less likely to have controlled glycemia (aRR = 0.75, 95% C.I: 0.58–0.97) compared to male participants. Homemakers were 51% less likely to have controlled glycemia (aRR = 0.49, 95% C.I: 0.23–1.01) compared to those that were employed. However, this association was not statistically significant. The following covariates were not associated with controlled glycemia: age, BMI, wealth index and tobacco use. (Table 4)

### Alcohol use and optimal treatment outcomes among those with multimorbidity

Among those with multimorbidity there was no difference in treatment outcomes for participants reporting alcohol use at least once a week and at least once a month (aRR = 1.00, C.I: 0.93–1.09) compared to those that reported no alcohol use. The associations were not statistically significant. (Table 5)

#### Other factors associated with optimal treatment outcomes among those with multimorbidity:

Among participants with multimorbidity those aged above 60 years were 6% less likely to have optimal treatment outcomes (aRR = 0.94, 95% C.I: 0.90–0.99) compared to those aged between 40 and 60 years. In addition, Immigrants (Mozambique or other) were 6% likely to have optimal treatment outcomes (aRR = 1.06, 95% C.I: 1.00–1.12) compared to those from South Africa. Obese (aRR = 0.89, 95% C.I: 0.84–0.94) and overweight (aRR = 0.92, 95% C.I: 0.89–0.97) were less likely to have optimal treatment outcomes compared to participants with a normal BMI. (Table 5)

### Sensitivity Analyses

We carried out a sensitivity analysis to determine the association between alcohol use and optimal treatment outcomes among those with multimorbidity. The results obtained across alcohol use categories were similar for both multimorbidity (HIV, hypertension and diabetes mellitus) (Table 5) and multimorbidity without HIV (Supplementary Tables 6).

## DISCUSSION

The study sought to establish the association between alcohol use and chronic diseases’ treatment outcomes (HIV viral load suppression, controlled hypertension, and glycemic control) among adults aged 40 years and above in rural South Africa. This study shows that the prevalence of optimal treatment outcome was highest for HIV and sub optimal (inadequate) for hypertension, diabetes and among those with multimorbidity. Furthermore, alcohol use was not significantly associated with any of the chronic diseases’ treatment outcomes (HIV, hypertension, diabetes mellitus and multimorbidity).

The prevalence of optimal hypertension treatment outcome was 42.7%, which is within the range of similar studies done in South Africa that reported a prevalence ranging from 19–56% of controlled hypertension outcomes (25). However, the WHO SAGE wave 2 reported that only 18% had controlled hypertension treatment outcome (6). We also reported a prevalence of 53.6% for optimal diabetes mellitus treatment outcome, which is much higher than a prevalence of 19% and 23% reported in previous studies done in South Africa (7)(21). The prevalence optimal HIV treatment outcome was 87.3%, which was similar to a population-based survey that reported 87% were virally suppressed(8). The prevalence of optimal hypertension and diabetes mellitus treatment outcomes are better than those reported in previous studies but still inadequate. The improved hypertension treatment outcome could be attributed to the improved health care amongst this population which has been followed up in the Agincourt HDSS since 1992.

Alcohol use was not associated with any of the chronic diseases (HIV, hypertension, and diabetes mellitus) treatment outcomes in this study population. These findings might have been influenced by several factors that include this study did not assess adherence to treatment for the participants that self-reported being on treatment. Baum et al, found that adherence to medication mediates the association between alcohol use and HIV treatment outcomes (26). In addition, this study population was an older population and Korhonen et al concluded that initiation of chronic disease medication was associated with a greater decline in alcohol consumption (27). Furthermore, a study done showed that alcohol consumption patterns differ with age and older age groups were associated with lower alcohol consumption (28). However, this study assessed the frequency of alcohol use and did not quantify the alcohol consumption of those that self-reported alcohol use. Lastly, alcohol use was self-reported in this study which could have resulted in social desirability bias thereby affecting our results. Stockwell et al found that infrequent drinkers under-estimate their consumption more than frequent drinkers (29). Recent studies have suggested using biomarkers such as Phosphatidyethanol, carbohydrate-deficient transferrin and gamma-glutamyl transferase to validate self-report alcohol use (30–32). Nonetheless, these biomarkers should be interpreted with caution in people with liver disease.

### Even though alcohol use was not associated with the chronic condition optimal treatment outcomes:

1. *For viral load suppression* this study findings are consistent with a study done in Kenya which showed that alcohol use was not associated with a suppressed viral load (15). Another study found that women aged 50 years and above were less likely to have a non-suppressed viral load than women under 50 years (33). Conversely, some studies that found a significant association between alcohol use and a high viral load were among a younger population and those that consumed more than 20 units of alcohol per week (12). Cook et al, suggested that differences in the way alcohol consumption is defined and measured can result in different conclusions on the relationship between alcohol consumption and HIV (34).
2. *For hypertension treatment outcomes*, findings are supported by a study done which found that older age groups had a better awareness of lifestyle changes and good treatment outcomes (16). A reduction in alcohol consumption was shown to reduce blood pressure among hypertensive men who were drinkers (13). Other studies showed light drinking to be protective and moderate to heavy drinking was associated with increased blood pressure, however, the effects of alcohol are heterogenous and vary according to dose and pattern (13).
3. *For diabetes mellitus treatment outcomes*, the findings are similar to the other studies in sub-Sahara Africa that showed alcohol was not associated with poor glycemic control (14)(17). However, other studies reported that moderate alcohol consumption of 5 to 20g of alcohol use was associated with better glycemic control as it decreases HbA1C and increases insulin sensitivity (30).
4. *For multimorbidity treatment outcomes*, there are a few studies that have looked at multimorbidity interventions and the evidence to support the different approaches is still limited (35). But a study done on hypertension control among people living with HIV-showed that alcohol use was not associated with the treatment outcomes (36).

In this study, some covariates such as age, sex and BMI were associated with treatment outcomes. Specifically, females were more likely than men to have optimal treatment outcomes for HIV and hypertension that is consistent with previous studies that reported females had better outcomes because they tend to adhere to treatment schedules more than men in South Africa (6).

Another important covariate in this study was age as such those older than 60 years were more likely to have optimal treatment outcomes for HIV, hypertension and diabetes mellitus, we attribute this to the impact of lifestyle modifications among older age groups following chronic disease diagnosis (27). On the other hand, those with multimorbidity had poorer treatment outcomes possibility due to challenges associated with multimorbidity, for example, polypharmacy leading to unfavorable drug side effects (3).

Overweight and obese participants had suboptimal treatment outcomes amongst those with multimorbidity due to increased risk of chronic conditions to overweight and obese participants (3–4).

It is important to consider the following limitations when interpreting this study’s findings. First, because this study was cross-sectional, it was not possible to determine the temporal relationship between alcohol use and treatment outcomes. Therefore, not possible to establish causality. Second, alcohol use was self-reported and that could be subjected to recall and social desirability bias. This could have led to underreporting and therefore biased the association of alcohol use and chronic diseases’ treatment outcomes towards the null. Lastly, the hypertension and blood glucose were assessed at one time point and therefore unable to determine/ establish long term control of hypertension and diabetes. For glycemic assessment, HbA1C assessment would have been a better indicator of glycemic control as it measures the average blood sugar levels over the past three months (14).

In conclusion, this study has shown that reported alcohol use is not associated with chronic diseases’ treatment outcomes (HIV, hypertension, diabetes mellitus and multimorbidity). In addition, the prevalence of optimal hypertension, diabetes mellitus and multimorbidity treatment outcomes were low. There is an urgent need to optimize treatment outcomes to reduce morbidity and mortality. We need to incorporate objective alcohol measurements in chronic diseases care settings. Furthermore, there is an urgent need to strengthen the management of hypertension and diabetes, by adopting the strategies deployed for HIV management. Also, critical to strengthen the management of multimorbidity in older populations.

## Availability of data and other materials

Data are available in a public, open access repository. Any additional data requests could be directed to chodziwadziwa.kabudula@wits.ac.za. The HAALSI baseline data are publicly available at the Harvard Centre for Population and Development Studies (HCPDS) programme website https://haalsi.org/data.

## Figures and Tables

**Table 1:** Baseline characteristics of HAALSI population (N = 5059) enrolled between November 2014 and November 2015.

Characteristic	Categories	n	%
Age Group (years)	40–60	2479	49.0
	61 and above	2580	51.0
Sex	Female	2714	53.7
	Male	2345	46.3
Country of origin	South Africa	3528	69.8
	Mozambique/other	1526	30.2
	No formal education	2306	45.7
Level of education	Some primary (1–7 years)	1716	34.0
	Some secondary (8–11 years)	574	11.4
	Secondary or more (12 + years)	446	8.9
	Never married	290	5.7
Marital status	Separated / divorced	650	12.9
	Widowed	1540	30.5
	Currently married	2575	50.9
	1	1046	20.7
	2	1001	19.8
Wealth asset index	3	991	19.6
	4	1007	19.9
	5	1014	20.0
	Living alone	534	10.6
Household size	Living with one other person	538	10.6
	Living in 3–6-person household	2438	48.2
	Living in 7 + person household	1549	30.6
	Underweight	258	5.5
BMI category	Normal	1719	36.7
	Overweight	1328	28.3
	Obese	1384	29.5
	Employed (part or full time)	805	16.0
Employment status	Not working	3719	73.7
	Homemaker	521	10.3
Tobacco use	Yes	447	8.4
	No	4608	91.6
	None	3887	76.9
Alcohol use	At least once a week	617	12.2
	At least once a month	551	10.9

The total number of observations for each variable does not add up to (N = 5059) in each column due to missing values.

Missing data for marital status: 4, alcohol use: 4, tobacco use: 4, country of origin: 5, employment status: 14, level of education: 17 and BMI: 370

**Table 2:** Association between reported alcohol use and HIV Viral load suppression amongst participants of the HAALSI study (N = 5059), enrolled between November 2014 and November 2015.

Characteristic	Categories	Total N(%)^a^	RR^b^ (95% C.I)	p value^d^	aRR^c^ (95% C.I)	p value^d^
	None	358 (89.5)	1	**0.080**	1	**0.438**
Alcohol Use	At least once a week	46 (80.7)	0.90 (0.79–1.02)		1.00 (0.93–1.09)	
	At least once a month	46 (79.3)	0.87 (0.77–1.02)		0.92 (0.80–1.05)	
Sex	Male	198 (82.2)	1	**0.001**	1	**0.296**
	Female	252 (92.0)	**1.12 (1.04–1.20)**		**1.13 (1.04–1.24)**	
Age group (in years)	40–60	312 (86.0)	1	**0.102**	1	**0.106**
	61 and above	138 (90.8)	1.06 (0.99–1.12)		1.06 (0.98–1.13)	
	No formal education	171 (86.4)	1	**0.097**	1	**0.045**
Education category	Some primary (1–7 years)	182 (89.7)	1.03 (0.97–1.12)		1.07 (0.98–1.16)	
	Some secondary (8–11 years)	60 (81.1)	0.94 (0.83–1.06)		0.98 (0.85–1.12)	
	Secondary or more (12 + years)	35 (94.6)	1.10 (0.99–1.20)		**1.14 (1.01–1.25)**	
	Never married	27 (77.1)	1	**0.099**	1	**0.058**
Marital status	Separated / divorced	93 (82.3)	1.07 (0.87–1.30)		1.02 (0.85–1.23)	0.798
	Widowed	156 (89.7)	1.16 (0.96–1.40)		1.08 (0.90–1.28)	0.399
	Currently married	174 (90.6)	1.17 (0.98–1.41)		1.16 (0.98–1.40)	0.092
	1	102 (83.6)	1	**0.230**	1	**0.510**
	2	102 (89.5)	1.07 (0.97–1.18)		1.07 (0.96–1.20)	
Wealth asset index	3	95 (91.3)	1.09 (0.99–1.2)		1.06 (0.95–1.18)	
	4	83 (83.0)	0.99 (0.88–1.12)		0.99 (0.87–1.13)	
	5	68 (90.7)	1.08 (0.90–1.20)		1.04 (0.92–1.18)	
	Underweight	38 (82.6)	0.98 (0.85–1.13)	**0.014**	0.98 (0.85–1.13)	**0.208**
BMI category	Normal	204 (84.3)	1		1	
	Overweight	110 (94)	**1.12 (1.03–1.20)**		1.06 (0.99–1.14)	
	Obese	87 (90.6	1.08 (0.98–1.16)		1.02 (0.93–1.11)	
Tobacco Use	Yes	32 (94.1)	1	**0.086**	1	**0.119**
	No	418 (86.9)	0.92 (0.84–1.01)		0.92 (0.81–1.02)	

C.I = confidence interval.

a Sample of each exposure category.

b Crude relative risk from modified Poisson regression.

c Adjusted relative risk from modified Poisson regression.

d The overall *P*-value for trend across all categories of individual variable. Significant values are in bold. 1 = > Reference category. Deviance Goodness of fit model = (108.498), p = 0.999

**Table 3:** Crude and adjusted association between alcohol use and controlled hypertension amongst participants of the HAALSI study (N = 5059), enrolled between November 2014 and November 2015.

Characteristic	Categories	Total N(%)^a^	RR^b^ (95% C.I)	p value^d^	aRR^c^ (95% C.I)	p value^d^
	None	613 (43.7)	1	**0.230**	1	**0.350**
Alcohol Use	At least once a week	43 (36.8)	0.84 (0.65–1.07)		0.88 (0.68–1.14)	
	At least once a month	57 (38.8)	0.89 (0.71–1.09)		0.88 (0.70–1.09)	
Sex	Male	238 (39.6)	1	**0.055**	1	**0.003**
	Female	475 (44.5)	1.12 (0.99–1.26)		**1.22 (1.07–1.39)**	
Age group (in years)	40–60	222 (38.1)	1	**0.006**	1	**0.007**
	61 and above	491 (45.3)	**1.18 (1.05–1.34)**		**1.18 (1.05–1.34)**	
Country of origin	South Africa	533 (43.7)	1	**0.201**	1	**0.343**
	Mozambique/other	179 (40.1)	0.92 (0.80–1.05)		0.93 (0.81–1.08)	
	1	114 (40.6)	1	**0.557**	1	**0.583**
	2	128 (41.6)	1.02 (0.84–1.23)		1.01 (0.83–1.23)	
Wealth asset index	3	129 (42.3)	1.04 (0.85–1.26)		1.00 (0.82–1.23)	
	4	175 (46.4)	1.14 (0.95–1.36)		1.13 (0.94–1.37)	
	5	167 (42.1)	1.03 (0.86–1.24)		1.08 (0.89–1.31)	
	Underweight	28 (53.8)	1.08 (0.82–1.42)	**0.005**	1.13 (0.85–1.49)	< 0.001
BMI category	Normal	188 (49.7)	1		1	
	Overweight	202 (43.1)	0.87 (0.74–1.00)		**0.84 (0.73–0.97)**	
	Obese	251 (39.5)	**0.79 (0.69–0.91)**		**0.75 (0.64–0.86)**	
Tobacco Use	Yes	80 (45.2)	1	**0.477**	1	**0.646**
	No	633 (42.5)	0.93 (0.79–1.11)		1.05 (0.86–1.27)	

C.I = confidence interval.

a Sample of each exposure category.

b Crude relative risk from modified Poisson regression.

c Adjusted relative risk from modified Poisson regression.

d The overall *P*-value for trend across all categories of individual variable. Significant values are in bold. 1 = > Reference category. Deviance Goodness of fit model = (1087.7), p = 0.990

**Table 4:** Crude and adjusted association between alcohol use and controlled glycemia amongst participants of the HAALSI study (N = 5059), enrolled between November 2014 and November 2015.

Characteristic	Categories	Total N(%)^a^	RR^b^ (95% C.I)	p value^d^	aRR^c^ (95% C.I)	p value^d^
Alcohol Use	None	128 (54.5)	1	**0.453**	1	**0.23**
	Yes	12 (46.2)	0.85 (0.55–1.3)		0.73 (0.44–1.22)	
Sex	Male	66 (58.9)	1	**0.135**	1	**0.027**
	Female	74 (49.7)	0.84 (0.67–1.05)		**0.75 (0.58–0.97)**	
Age group (in years)	40–60	43 (45.7)	1	**0.067**	1	**0.182**
	61 and above	97 (68.1)	1.27 (0.98–1.63)		1.22 (0.91–1.64)	
	Employed	12 (46.2)	1	**0.033**	1	**0.026**
Employment status	Not working	120 (58.3)	1.26 (0.82–1.94)		1.10 (0.67–1.82)	
	Homemaker	8 (27.6)	0.6 (0.29–1.23)		0.49 (0.23–1.01)	
	1	16 (51.6)	1	**0.754**	1	**0.43**
	2	19 (45.2)	0.88 (0.54–1.41)		0.93 (0.55–1.60)	
Wealth asset index	3	29 (56.9)	1.10 (0.73–1.67)		1.16 (0.71–1.89)	
	4	34 (58.6)	1.14 (0.76–1.70)		1.36 (0.86–2.20)	
	5	42 (53.2)	1.03 (0.69–1.50)		1.11 (0.70–1.76)	
	Underweight	2 (100)	**1.85 (1.42–2.40)**		**2.45 (1.20–4.97)**	**0.8**
BMI category	Normal	26 (54.2)	1		1	
	Overweight	36 (49.3)	**0.91 (0.87–0.97)**		0.92 (0.65–1.31)	
	Obese	57 (51.8)	**0.89 (0.84–0.93)**		1.01 (0.73–1.42)	
Tobacco Use	Yes	13 (61.9)	1	**0.389**	1	**0.547**
	No	127 (52.9)	0.85 (0.60–1.22)		0.86 (0.54–1.39)	

C.I = confidence interval.

a Sample of each exposure category.

b Crude relative risk from modified Poisson regression.

c Adjusted relative risk from modified Poisson regression.

d The overall *P*-value for trend across all categories of individual variable. Significant values are in bold. 1 = > Reference category. Deviance Goodness of fit model = (147.51), p = 1.00

**Table 5:** Crude and adjusted association between alcohol use and optimal treatment outcomes among those with multimorbidity amongst participants of the HAALSI study (N = 5059), enrolled between November 2014 and November 2015.

Characteristic	Categories	Total N(%)^a^	RR^b^ (95% C.I)	p value^d^	aRR^c^ (95% C.I)	p value^d^
	None	382 (51.6)	1	0.357	1	0.981
Alcohol Use	At least once a week	50 (59.5)	1.05 (0.98–1.15)		1.00 (0.93–1.09)	
	At least once a month	43 (52.4)	1.00 (0.93–1.08)		1.00 (0.93–1.08)	
Sex	Male	190 (50.5)	1	0.352	1	0.296
	Female	285 (53.7)	1.02 (0.98–1.07)		1.03 (0.97–1.08)	
Age group (in years)	40–60	265 (58.4)	1	**< 0.001**	1	**0.013**
	61 and above	210 (46.4)	**0.92 (0.89–0.96)**		**0.94 (0.90–0.99)**	
Country of origin	South Africa	329 (49.9)	1	**0.014**	1	**0.040**
	Mozambique/other	145 (58.9)	**1.06 (1.01–1.11)**		**1.06 (1.0–1.12)**	
	No formal education	209 (52.1)	1	0.174	1	0.173
Education category	Some primary (1–7 years)	171 (52.8)	1.00 (0.96–1.05)		1.02 (0.97–1.08)	
	Some secondary (8–11 years)	64 (59.8)	1.05 (0.98–1.12)		1.08 (0.99–1.16)	
	Secondary or more (12 + years)	31 (43.1)	0.94 (0.86–1.03)		0.99 (0.89–1.08)	
	Never married	19 (52.8)	1	**0.001**	1	0.026
	Separated / divorced	96 (65.8)	1.08 (0.97–1.22)		1.01 (0.96–1.20)	
Marital status	Widowed	168 (52.3)	0.99 (0.89–1.12)		1.03 (0.92–1.15)	
	Currently married	192 (47.6)	0.97 (0.86–1.08)		0.98 (0.87–1.09)	
	1	97 (61.4)	1	**0.030**	1	0.884
	2	93 (54.1)	0.95 (0.89–1.04)		0.97 (0.90–1.04)	
Wealth asset index	3	90 (52.6)	0.95 (0.88–1.01)		0.98 (0.92–1.06)	
	4	106 (51.5)	0.94 (0.88–1.00)		0.98 (0.90–1.05)	
	5	89 (44.5)	**0.89 (0.84–0.96)**		0.96 (0.89–1.04)	
	Underweight	23 (74.2)	1.06 (0.96–1.17)	**< 0.001**	1.04 (0.94–1.16)	**0.001**
BMI category	Normal	164 (64.3)	1		1	
	Overweight	126 (50.2)	**0.91 (0.87–0.97)**		**0.92 (0.87–0.97)**	
	Obese	142 (45.5)	**0.89 (0.84–0.93)**		**0.89 (0.84–0.94)**	
Tobacco Use	Yes	45 (56.3)	1	0.459	1	0.742
	No	430 (52.0)	0.97 (0.90–1.05)		0.99 (0.91–1.07)	

C.I = confidence interval.

a Sample of each exposure category.

b Crude relative risk from modified Poisson regression.

c Adjusted relative risk from modified Poisson regression.

d The overall *P*-value for trend across all categories of individual variable. Significant values are in bold. 1 = > Reference category. Deviance Goodness of fit model = (131.66), p = 1.00

## Appendix

Appendix 3 and 4 are not available with this version.

## Abbreviations

AHDSS: Agincourt Health and Socio-demographic Surveillance Site
AIDS: Acquired Immuno-Deficiency Syndrome
ART: Anti Retro-viral Therapy
BMI: Body Mass Index
C.W.K: Chodziwadziwa W. Kabudula
CVDs: Cardiovascular diseases
HAALSI: The Health and Ageing in Africa – a Longitudinal Study in an INDEPTH community
HbA1C: Hemogloblin A1C
HIV: Human Immunodeficiency Virus
J.M.F: Joel Msafiri Francis
NCDs: Non-communicable diseases
PLHIV: People Living with HIV
R.M: Rumbidzai Mupfuti
SA NSP-NCD: South African National Strategic Plan for the Prevention and Control of Non-communicable diseases
SADHS: South African Demographic and Health Survey
WHO-SAGE: World Health Organisation Study on Global AGEing and Adult Health
WHO: World Health Organisation

## References

[1] Peer N, de Villiers A, Jonathan D, Kalombo C, Kengne AP. Care and management of a double burden of chronic diseases: Experiences of patients and perceptions of their healthcare providers. PLoS One. 2020 Jul 1;15(7 July). 10.1371/journal.pone.0235710 PMC7365408 32673339

[2] Jardim TV, Reiger S, Abrahams-Gessel S, Gomez-Olive FX, Wagner RG, Wade A, Hypertension management in a population of older adults in rural South Africa. J Hypertens. 2017;35(6):1283–9. 28441697 10.1097/HJH.0000000000001312 PMC5505070

[3] Basto-Abreu A, Barrientos-Gutierrez T, Wade AN, Oliveira de Melo D, Semeão de Souza AS, Nunes BP, Multimorbidity matters in low and middle-income countries. Journal of Multimorbidity and Comorbidity [Internet]. 2022 Jan 16;12:263355652211060. Available from: http://journals.sagepub.com/doi/10.1177/26335565221106074 10.1177/26335565221106074 PMC9208045 35734547

[4] Minja NM, Nakagaayi D, Aliku T, Zhang W, Ssinabulya I, Nabaale J, Cardiovascular diseases in Africa in the twenty-first century: Gaps and priorities going forward. Vol. 9, Frontiers in Cardiovascular Medicine. Frontiers Media S.A.; 2022. 10.3389/fcvm.2022.1008335 PMC9686438 36440012

[5] Aboderin I. Africa Aging: 2020 International Population Reports [Internet]. Available from: https://www.researchgate.net/publication/344138372

[6] Ware LJ, Chidumwa G, Charlton K, Schutte AE, Kowal P. Predictors of hypertension awareness, treatment and control in South Africa: results from the WHO-SAGE population survey (Wave 2). J Hum Hypertens. 2019 Feb 1;33(2):157–66. 30382179 10.1038/s41371-018-0125-3

[7] South Africa National NCD Strategic Plan NATIONAL STRATEGIC PLAN FOR THE PREVENTION AND CONTROL OF NON-COMMUNICABLE DISEASES 2020–2025.

[8] Zuma K, Simbayi L, Zungu N, Moyo S, Marinda E, Jooste S, The HIV Epidemic in South Africa: Key Findings from 2017 National Population-Based Survey. Int J Environ Res Public Health. 2022 Jul 1;19(13). 10.3390/ijerph19138125 PMC9265818 35805784

[9] World Health Organization. Noncommunicable diseases country profiles 2018 [Internet]. Geneva: World Health Organization; 2018 [cited 2022 Oct 18]. 223

[10] Crawford TN, Thornton AC. Alcohol Use and Multimorbidity Among Individuals Living with HIV. AIDS Behav. 2019 Jan 15;23(1):152–60. 30088200 10.1007/s10461-018-2242-y

[11] Micklesfield LK, Kolkenbeck-Ruh A, Mukoma G, Prioreschi A, Said-Mohamed R, Ware LJ, The Healthy Aging Adult South Africa report card: a systematic review of the evidence between 2013 and 2020 for middle-aged South African men and women. Vol. 33, Cardiovascular journal of Africa. NLM (Medline); 2022. p. 200–19. 35789240 10.5830/CVJA-2022-015 PMC9650148

[12] Dahab M, Charalambous S, Karstaedt AS, Fielding KL, Hamilton R, Grange L La, Contrasting predictors of poor antiretroviral therapy outcomes in two South African HIV programmes: a cohort study [Internet]. 2010. Available from: http://www.biomedcentral.com/1471-2458/10/430 10.1186/1471-2458-10-430 PMC2920888 20649946

[13] Ojangba T, Boamah S, Miao Y, Guo X, Fen Y, Agboyibor C, Comprehensive effects of lifestyle reform, adherence, and related factors on hypertension control: A review. Journal of Clinical Hypertension. John Wiley and Sons Inc; 2023. 10.1111/jch.14653 PMC10246465 37161520

[14] Bitew ZW, Alemu A, Jember DA, Tadesse E, Getaneh FB, Sied A, Prevalence of Glycemic Control and Factors Associated With Poor Glycemic Control: A Systematic Review and Meta-analysis. Inquiry. 2023 Jan 1;60. 10.1177/00469580231155716 PMC10071101 36852627

[15] Long JE, Richardson BA, Wanje G, Wilson KS, Shafi J, Mandaliya K, Alcohol use and viral suppression in HIVpositive Kenyan female sex workers on antiretroviral therapy. PLoS One. 2020 Nov 1;15(11 November). 10.1371/journal.pone.0242817 PMC7685481 33232378

[16] Kayima J, Wanyenze RK, Katamba A, Leontsini E, Nuwaha F. Hypertension awareness, treatment and control in Africa: A systematic review. BMC Cardiovasc Disord. 2013 Aug 2;13. 10.1186/1471-2261-13-54 PMC3750220 23915151

[17] Fiseha T, Alemayehu E, Kassahun W, Adamu A, Gebreweld A. Factors associated with glycemic control among diabetic adult out-patients in Northeast Ethiopia. BMC Res Notes. 2018 May 18;11(1). 10.1186/s13104-018-3423-5 PMC5960206 29776447

[18] Kahn K, Collinson MA, Gómez-Olivé FX, Mokoena O, Twine R, Mee P, Afolabi SA, Clark BD, Kabudula CW, Khosa A, Khoza S. Profile: Agincourt health and socio-demographic surveillance system. International journal of epidemiology. 2012 Aug 1;41(4):988–1001 22933647 10.1093/ije/dys115 PMC3429877

[19] ART Clinical Guidelines for the Management of HIV in Adults, Pregnancy, Adolescents, Children, Infants and Neonates.

[20] Seedat YK, Rayner BL, Veriava Y. South African hypertension practice guideline 2014. Cardiovasc J Afr. 2014 Nov 1;25(6):288–94. 25629715 10.5830/CVJA-2014-062 PMC4327181

[21] Masilela C, Pearce B, Ongole JJ, Adeniyi OV, Benjeddou M. Factors associated with glycemic control among South African adult residents of Mkhondo municipality living with diabetes mellitus. Medicine. 2020 Nov 25;99(48):e23467. 33235135 10.1097/MD.0000000000023467 PMC7710224

[22] Dyussenbayev A. Age Periods Of Human Life. Adv Soc Sci Res J. 2017 Mar 25;4(6).

[23] StataCorp. 2019. Stata Statistical Software: Release 16. College Station TSL. Stata | StataCorp LLC [Internet]. [cited 2021 Apr 12]. Available from: https://www.stata.com/company/. Stata.

[24] Yelland LN, Salter AB, Ryan P. Performance of the modified poisson regression approach for estimating relative risks from clustered prospective data. Am J Epidemiol. 2011 Oct 15;174(8):984–92. 21841157 10.1093/aje/kwr183

[25] Masilela C, Pearce B, Ongole JJ, Adeniyi OV, Benjeddou M. Cross-sectional study of prevalence and determinants of uncontrolled hypertension among South African adult residents of Mkhondo municipality. BMC Public Health. 2020 Jul 6;20(1). 10.1186/s12889-020-09174-7 PMC7339580 32631300

[26] Baum MK, Rafie C, Lai S, Sales S, Bryan Page J, Campa A. Alcohol Use Accelerates HIV Disease Progression. 10.1089/aid.2009.0211 PMC2875959 20455765

[27] Korhonen MJ, Pentti J, Hartikainen J, Ilomäki J, Setoguchi S, Liew D, Lifestyle Changes in Relation to Initiation of Antihypertensive and Lipid-Lowering Medication: A Cohort Study. J Am Heart Assoc. 2020 Feb 18;9(4). 10.1161/JAHA.119.014168 PMC7070189 32019405

[28] Lesko CR, Nance RM, Lau B, Fojo AT, Hutton HE, Delaney JAC, Changing Patterns of Alcohol Use and Probability of Unsuppressed Viral Load Among Treated Patients with HIV Engaged in Routine Care in the United States. AIDS Behav. 2021 Apr 1;25(4):1072–82. 33064249 10.1007/s10461-020-03065-z PMC7979457

[29] Livingston M, Callinan S. Underreporting in alcohol surveys: whose drinking is underestimated? J Stud Alcohol Drugs. 2015 Jan;76(1):158–64. 25486405

[30] Pastor A, Conn J, MacIsaac RJ, Bonomo Y. Alcohol and illicit drug use in people with diabetes. Vol. 8, The Lancet Diabetes and Endocrinology. Lancet Publishing Group; 2020. p. 239–48. 31958403 10.1016/S2213-8587(19)30410-3

[31] Hahn JA, Murnane PM, Vittinghoff E, Muyindike WR, Emenyonu NI, Fatch R, Factors associated with phosphatidylethanol (PEth) sensitivity for detecting unhealthy alcohol use: An individual patient data meta-analysis. Alcohol Clin Exp Res. 2021 Jun 1;45(6):1166–87. 33837975 10.1111/acer.14611 PMC8254773

[32] Francis JM, Weiss HA, Helander A, Kapiga SH, Changalucha J, Grosskurth H. Comparison of self-reported alcohol use with the alcohol biomarker phosphatidylethanol among young people in northern Tanzania. Drug Alcohol Depend. 2015 Nov 1;156:289–96. 26455816 10.1016/j.drugalcdep.2015.09.027

[33] Myers B, Lombard C, Joska JA, Abdullah F, Naledi T, Lund C, Associations Between Patterns of Alcohol Use and Viral Load Suppression Amongst Women Living with HIV in South Africa. AIDS Behav. 2021 Nov 1;25(11):3758–69. 33876383 10.1007/s10461-021-03263-3 PMC8560660

[34] Richards VL, Leeman RF, Wang Y, Cook C, Prins C, Ennis N, Identifying the best measures of alcohol consumption to predict future HIV viral suppression trajectories. AIDS Behav. 2022 Oct 1;26(10):3242–53. 35380289 10.1007/s10461-022-03674-w PMC9474662

[35] Skou ST, Mair FS, Fortin M, Guthrie B, Nunes BP, Miranda JJ, Multimorbidity. Nat Rev Dis Primers. 2022 Dec 1;8(1). 10.1038/s41572-022-00376-4 PMC7613517 35835758

[36] Mutemwa M, Peer N, De Villiers A, Mukasa B, Matsha TE, Mills EJ, Prevalence, detection, treatment, and control of hypertension in human immunodeficiency virus (HIV)-infected patients attending HIV clinics in the Western Cape Province, South Africa. Medicine (United States). 2018 Aug 1;97(35). 10.1097/MD.0000000000012121 PMC6392528 30170445

